# The effects of lowering barometric pressure on pain behavior and the stress hormone in mice with neuropathic pain

**DOI:** 10.1371/journal.pone.0317767

**Published:** 2025-01-17

**Authors:** Yuki Terajima, Jun Sato, Hideaki Inagaki, Takahiro Ushida

**Affiliations:** 1 Department of Pain Medicine and Pain Relief Surgery, Medical Center, Aichi Medical University, Nagakute, Aichi, Japan; 2 Department of Pain Medicine, Aichi Medical University, Nagakute, Aichi, Japan; 3 Department of Physical Therapy, College of Life and Health Sciences, Chubu University, Kasugai, Aichi, Japan; University of Minho, PORTUGAL

## Abstract

**Background:**

Lowering barometric pressure (LP) can exacerbate neuropathic pain. However, animal studies in this field are limited to a few conditions. Furthermore, although sympathetic involvement has been reported as a possible mechanism, whether the sympathetic nervous system is involved in the hypothalamic-pituitary-adrenal (HPA) axis remains unknown. To address these issues, we investigated LP-induced hyperalgesia by focusing on the cumulative effect of LP and measuring plasma corticosterone levels as a marker of HPA axis activation in mice.

**Methods:**

Mice with chronic constriction injury (CCI) were used in this study. For behavioral tests, two types of LP stimulation were adopted: a single LP at 20 hPa (Single LP) and three consecutive LPs at 20 hPa (3LPs). Twelve mice were used for each protocol. The no-pressure-change protocol was used as the control. The mechanical sensitivity was tested before and after LP stimulation using von Frey filaments (vF). For corticosterone measurements, six CCI and six intact mice were exposed to 3LPs (CCI-3LPs and INT-3LPs), and another six CCI and six intact mice were exposed to the no-pressure-change protocol (CCI-NP and INT-NP). Blood samples were collected immediately after exposure. Plasma corticosterone levels were measured by ELISA.

**Results:**

The number of paw elevations by vF before and after LP stimulation did not differ significantly in either the Single LP or the no-pressure-change protocol. For the 3LPs, the number of paw elevations after LP stimulation was significantly greater than before stimulation. Plasma corticosterone levels in the CCI-3LPs were significantly higher than those in CCI-NPs. In intact mice, there was no significant difference in plasma corticosterone levels between the INT-3LPs and INT-NPs.

**Conclusions:**

LP has a cumulative effect on neuropathic pain. Hypothalamic-pituitary-adrenal axis activation may have an important relationship with LP-induced pain in mice.

## Introduction

It is well-known that weather can trigger or worsen various types of pain. Atmospheric pressure (AP) is a meteorological factor that affects pain. Clinical studies have shown that changes in atmospheric pressure can cause various types of pain, including migraines [1], rheumatoid arthritis [2], fibromyalgia [3], musculoskeletal pain (osteoarthritis-induced pain [4] and back pain [5]), and neuropathic pain (phantom pain [6] and spinal cord injury [7]). Neuropathic pain is caused by damage to the somatosensory system [8] and is characterized by intense pain. In chronic conditions, the pathogenesis is complex, and the outcomes tend to be poor. Despite these reports, there is little scientific consensus regarding the association between atmospheric pressure and pain. Recent reviews on the association between weather and pain have highlighted the challenges in clinical studies of weather-related pain owing to heterogeneity [9,10]. There are several weather parameters (temperature, humidity, and pressure) that make it difficult to investigate the effects of each parameter separately in the real world. Additionally, it has been suggested that visual and audible information may influence participants perception [11], especially when they are aware of the study’s objectives [12]. In contrast, animal studies can be conducted under various environmental conditions and are unaffected by judgment bias. Furthermore, it is essential to elucidate the mechanisms by which changes in atmospheric pressure exacerbate pain. The results of animal experiments would provide strong evidence and new insights into the relationship between atmospheric pressure and pain. However, these studies have been scarce.

Animal studies have shown that atmospheric pressure can affect pain. Sato et al. reported that lowering barometric pressure exaggerated mechanical hypersensitivity in rats with neuropathic pain [13] and induced arthritis [14]. Additionally, they reported that the rate and magnitude of LP can aggravate pain behavior in rats with neuropathic pain [15]. However, these studies were based on experiments conducted under a limited number of conditions. Further experiments using a greater variety of experimental conditions will provide more robust scientific evidence in this field. The repeated stimulation of changes in barometric pressure may exacerbate the effects on pain. However, to the best of our knowledge, no animal experiments have been conducted to date. In terms of animal species, almost all previous studies in this field used rats, which are excellent for behavioral experiments and morphological evaluations. However, they have the disadvantage of being difficult to use in experiments involving transgenesis methods. Experiments using mice are expected to contribute to the development of this field, as they can be used in experiments involving genetic methods. However, no studies have reported an association between atmospheric pressure and pain in mice.

Sympathetic nervous system activity may play an important role in the mechanisms underlying the interaction between barometric pressure and pain [16]. Lowering barometric pressure activates the autonomic nervous system in rats [17]. Moreover, neuropathic pain-related behavior in rats was aggravated by LP, and this effect was blocked by sympathectomy [13]. These results suggest that the mechanisms that increase sympathetic activity may parallel those that contribute to the development of meteoropathy. It has also been suggested that changes in HPA axis activation in response to neuropathic pain may play an important role in the relationship between barometric pressure and pain. However, it remains unknown whether changes in barometric pressure activate the HPA axis.

In the present study, we investigated the effects of LP on mechanical hypersensitivity in mice with neuropathic pain. Moreover, we focused on the cumulative effect of pressure changes and examined whether it could be a significant parameter of barometric pressure that affects pain. In addition, we measured the plasma corticosterone levels as a marker of HPA axis activation in mice. The findings of this study can provide clinicians with important evidence to explain changes in pressure-related neuropathic symptoms. In addition, it contributes to the development of future therapeutic targets by advancing our understanding of their pathophysiology.

## Materials and methods

### Animals

All experiments were performed in accordance with the Guidelines for Animal Experiments of Aichi Medical University, Directive 2010/63/EU of the European Parliament on the protection of animals used for scientific purposes, and the IASP Guidelines for the Use of Animals in Research. All animal experiments were approved by the Animal Experiment Committee of Aichi Medical University (No. 2020–108, 2021–11, 2022–103). We used 48 female C57BL/6J mice (10 weeks old at the beginning of the experiments) obtained from Japan SLC, Inc. (Shizuoka, Japan). They were housed in an environment with a mean temperature of 24±1°C, mean humidity of 50±10%, and 12/12 h light-dark cycles (light on at 7:45 a.m.). They had access to food and water *ad libitum* and were acclimated for at least one week before the experiments. All surgeries were performed using an intraperitoneal injection of a mixture of anesthetic agents [18]. Every effort was made to minimize suffering during and after surgery. At the endpoint (three weeks after surgery), mice used in behavioral tests were euthanized with carbon dioxide with an appropriate pressure regulator and flow meter, while mice used for corticosterone measurement were sacrificed through decapitation with sharp blades.

### CCI surgery

Chronic constriction injuries were induced as described in an earlier report on constriction of the sciatic nerve [19]. The surgical procedure consisted of the following steps: mice were injected intraperitoneally with a combination of medetomidine (0.3 mg/kg), midazolam (4 mg/kg), and butorphanol (5 mg/kg) diluted in a saline solution (2.9 mL/kg) [18]. Under deep anesthesia, a 5-mm incision was made in the right mid-thigh to expose the sciatic nerve. The sciatic nerve was loosely tied to a 4–0 chromic gut (Johnson & Johnson, New Brunswick, NJ, USA) at three points, and the distance between the adjacent ligatures was approximately 1 mm. Postoperatively, the mice were quickly awakened with atipamezole (3.0 mg/kg) diluted in saline solution (4.4 mL/kg). We then checked whether paralysis had occurred in the right hind paws of any of the mice.

### Assessment of mechanical sensitivity after CCI

To acclimatize the mice to the test environment, they were placed in their test cages for 30 min the day before the experiments. The acclimatization time was set to 20 minutes. If the animal did not settle, the acclimatization time was increased by 10 minutes. Test cages with wire mesh floors were used to access glabrous skin on the plantar surface of the right hind paw. The sensitivity to punctate mechanical stimuli was determined using von Frey filaments with strengths of 0.40, 0.70, and 1.6 mN (Aesthesio ®CE, DanMic Global, LLC, San Jose, CA, USA). Each filament was applied to the center of the right hind paw ten times, with an interval of 3–5 s between applications [20,21]. The application was performed in increasing order of strength, with intervals of at least 1 minute between each application. The number of paw elevations was counted. In this study, we selected these filaments based on preliminary results and experimental constraints. In normal mice, all three selected filaments are generally considered non-noxious, as evidenced by low response rates. After CCI surgery, we observed significant increases in responses to these filaments, which we interpreted as the development of allodynia. Therefore, these filaments were adequate for detecting allodynia.

### Lowering barometric pressure

In the present experiment, we used a pressure-controlled climatic chamber that could lower barometric pressure at various rates and ranges [15]. The chamber can maintain its barometric pressure independent of atmospheric pressure changes. Two types of LP protocols were used in the present study: a single LP and three consecutive LPs (3LPs). In the single LP protocol, the barometric pressure in the climate chamber was lowered from atmospheric pressure by 20 hPa in 5 minutes at the beginning of each experiment and then immediately returned to the normal level within 5 minutes. In the 3LPs protocol, a single LP was repeated three times without pause.

### Plasma corticosterone levels

Plasma samples were collected from CCI and age-matched, intact mice. Animals were decapitated, blood was collected, and EDTA2Na was added as an anticoagulant (1 mg/mL). The samples were centrifuged at 1700×g for 15 minutes at 4°C, and the supernatant was collected. Plasma was stored at −80°C until corticosterone was measured. Plasma corticosterone levels were measured using ELISA kits in duplicate. In the present study, we used a dedicated ELISA kit (corticosterone: Invitrogen Corticosterone Competitive ELISA Kit, Thermo Fisher Scientific, Waltham, MA, USA). The lowest detection limit was 18.6 pg/mL. The inter- and intra-assay coefficient of variation (CV) values were 7.9% and 5.2%, respectively. The samples were mixed with the dissociation reagent at a 1:1 ratio. Fifty microliters of the standard or samples diluted with the assay buffer were added to the appropriate wells. Assay buffer (75 μL) was added to detect non-specific binding (NSB). Then, 25 μL of corticosterone conjugate was added to each well, and 25 μL of corticosterone antibody was added to all wells except NSB. The plates were then incubated for 1 hour at room temperature (24±1°C) with shaking. After the plates were washed, 100 μL of TMB (3,3′,5,5′-tetramethylbenzidine) substrate was added to each well, and the plates were incubated for 30 minutes at room temperature. Then, 50 μL of stop solution was added to each well. The absorbance of the plate was measured at 450 nm within 10 minutes of the addition of the stop solution.

### Experiment 1: Effects of LP on mechanical hyperalgesia in CCI mice

**Fig 1:**
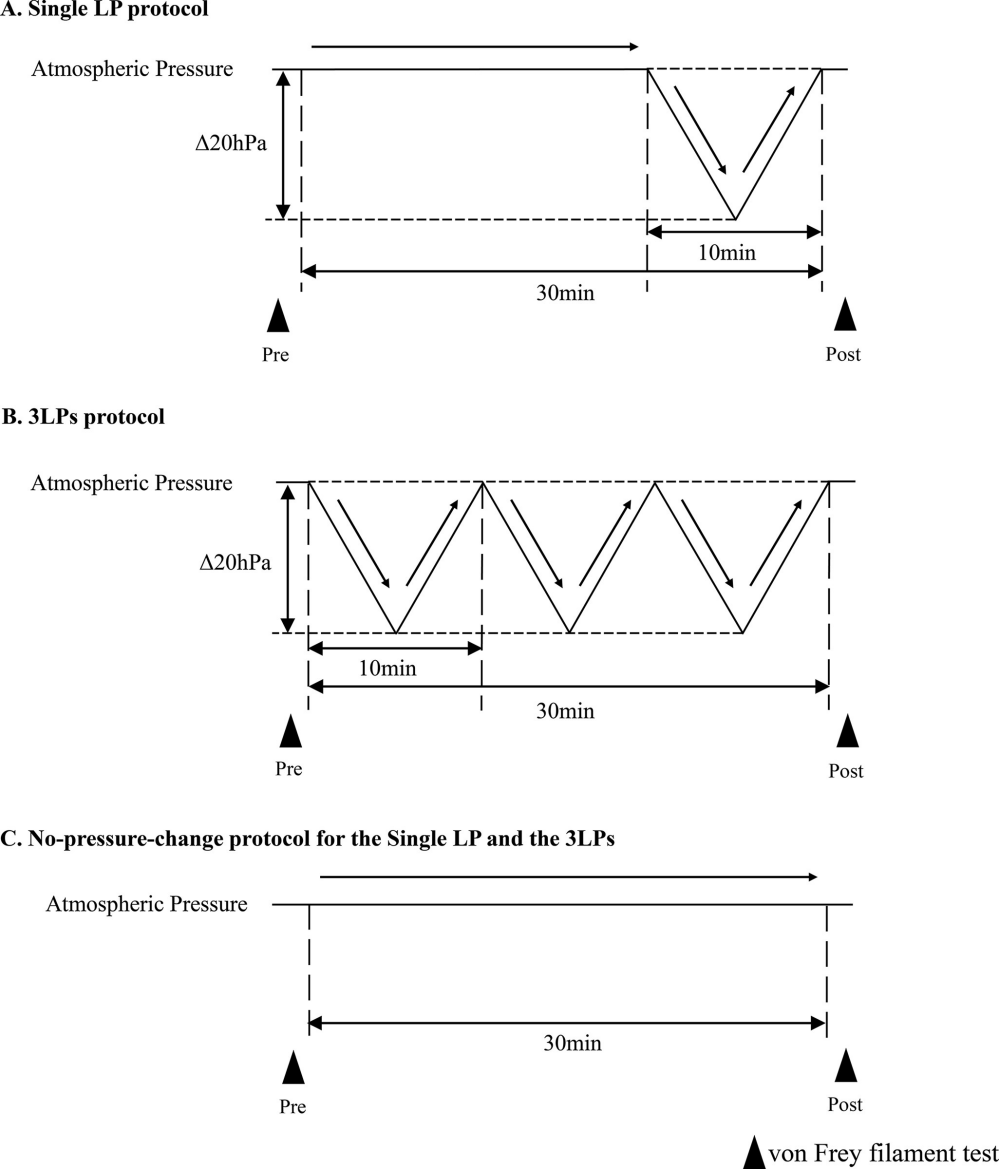
Protocol for lowering barometric pressure for behavioral tests. This figure shows the protocols for lowering barometric pressure in behavioral tests. The solid black line in the figure indicates the barometric pressure in the climate chamber. The arrows indicate the timing of the von Frey filament test.

A total of 24 CCI mice, 12 mice in the Single LP group and another 12 mice in the 3LPs group, were used in this behavioral experiment. To confirm that the mice had mechanical hypersensitivity following CCI surgery, their sensitivity to mechanical stimuli was assessed weekly for up to three weeks using three different thicknesses of von Frey filaments. All mice exhibiting a greater number of paw elevations three weeks after CCI surgery than those before CCI surgery were included in the following step. Sensitivity to punctate mechanical stimuli was determined using von Frey filaments before and after LP exposure (Fig 1). Control protocols without pressure changes (no-pressure-change protocols) were performed for each mouse exposed to either the single LP or 3LPs. In the pressure-control protocol, the barometric pressure in the climate chamber was maintained at atmospheric pressure throughout the experiment. The LP protocols (the single LP and the 3LPs) and the no-pressure-change protocols were performed on each mouse over one of two consecutive days. The investigator performing the behavioral tests did not know which pressure protocol was being administered on either day.

For each protocol, the mice underwent pre-tests after 20 minutes of acclimation to the climate chamber. Post-tests were conducted immediately after LP stimulation or the no-pressure-change protocol. In the present study, we maintained a 30-minute interval between the pre-test and post-test for each protocol (Fig 1). The Single LP protocol involved an additional 20-minute period at the initial atmospheric pressure, followed by one episode of LP at 20 hPa for a total of 30 minutes (Fig 1A). The 3LPs protocol included three consecutive episodes of LP at 20 hPa, starting from the initial outside pressure, which totaled 30 minutes (Fig 1B). In the no-pressure-change protocol for both the single LP and 3LPs, the barometric pressure in the climate chamber was maintained at the initial outside atmospheric pressure for 30 minutes (Fig 1C).

### Experiment 2: Effects of LP on plasma corticosterone levels in CCI mice

**Fig 2:**
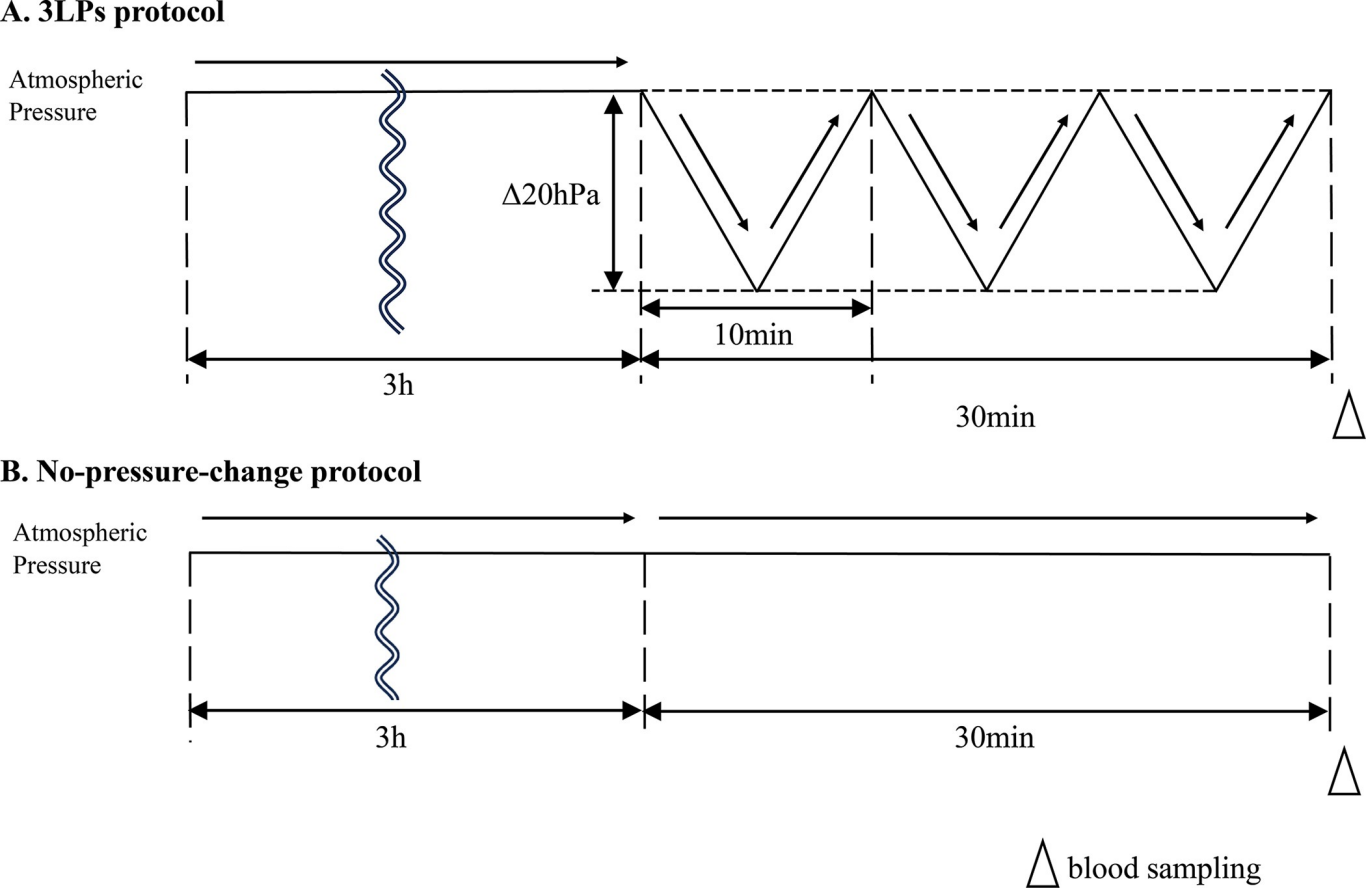
Protocol for lowering the barometric pressure for plasma corticosterone measurement. This figure shows the protocols for lowering barometric pressure for plasma corticosterone measurement. The solid black line in the figure indicates the barometric pressure in the climate chamber. Arrows indicate the timing of the blood sampling.

Twenty-four mice (12 CCI and 12 intact) were used in this experiment. As plasma corticosterone returned to normal levels within 3 hours after manipulation [22], the acclimation time in this experiment was set at 3 hours (Fig 2). Six CCI mice and six intact mice were exposed to 3LPs (CCI-3LPs and INT-3LPs) (Fig 2A). Six CCI and six intact mice were maintained in the climate chamber at atmospheric pressure throughout the experiment (CCI-NP and INT-NP) (Fig 2B). After each exposure, the mice were immediately decapitated, their blood was collected, and EDTA2Na was added as an anticoagulant (1 mg/mL). To mitigate the impact of the circadian rhythm of plasma corticosterone levels, blood collection was scheduled between 15:00 and 16:00.

### Statistics

Data were analyzed using SPSS Statistics version 28.0.1.1(14) (IBM, Armonk, New York, NY, USA). The results of the behavioral tests are presented as the mean ± standard error (SEM), and the ELISA results are expressed as the mean value. The mechanical sensitivity between Single LP and 3LP mice was compared using the Mann–Whitney U test. Behavioral tests in Experiment 1 were conducted using the Wilcoxon signed-rank sum test. The ELISA results were compared using the Kruskal–Wallis test to evaluate differences among more than two groups. If the Kruskal–Wallis test revealed significant differences (*P* < 0.5), post-hoc pairwise comparisons were performed using the Mann–Whitney U test. To account for multiple comparisons, *P*-values were adjusted using the Bonferroni correction method.

## Results

### Experiment 1: Effects of LP on mechanical hyperalgesia in CCI mice

**Fig 3:**
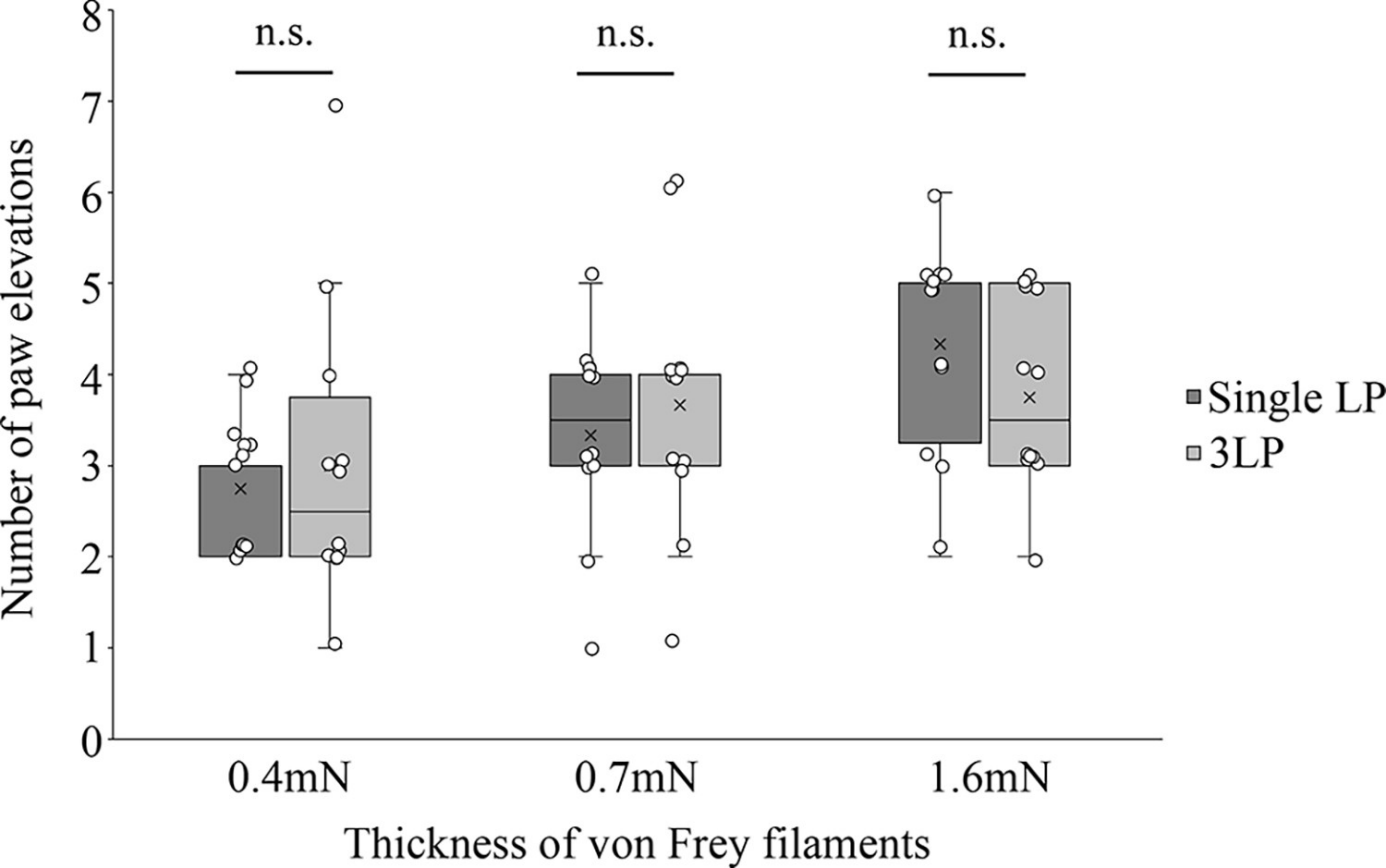
Mechanical hyperalgesia in CCI mice for single LP and 3LPs after CCI surgery. The box-and-whisker plot shows the comparison between mechanical hyperalgesia in mice for the Single LP or the 3LPs three weeks after CCI surgery. The notches in the box plots indicate the median, with the interquartile range being the difference between the third and first quartiles. Lower and upper whiskers indicate the minimum and maximum points within a distance of 1.5 times the interquartile range from the lower or upper quartile. All values were plotted (n = 12 per group). The number of paw elevations between mice for the Single LP and 3LPs were compared using the Mann–Whitney U Test. n.s.; Not Significant. Bar; SEM.

Mechanical hyperalgesia was observed in all mice in the LP and 3LP groups after CCI surgery. Mechanical hyperalgesia persisted for at least three weeks in both groups of mice for the Single LP and 3LPs. In our preliminary experiments, hyperalgesia in CCI mice peaked two weeks after CCI surgery, which is consistent with previous reports [23,24]. Mechanical sensitivities varied significantly two weeks after CCI surgery; however, this variance decreased by the third week. Thus, we decided to perform subsequent experiments three weeks after CCI surgery. At this time point, there was no significant difference in the number of paw elevations in response to all three filament thicknesses between mice subjected to the single LP and those subjected to the 3LPs (Fig 3) (0.4 mN, *P* = .932; 0.7 mN, *P* = .630; 1.6 mN, *P* = .219).

**Fig 4:**
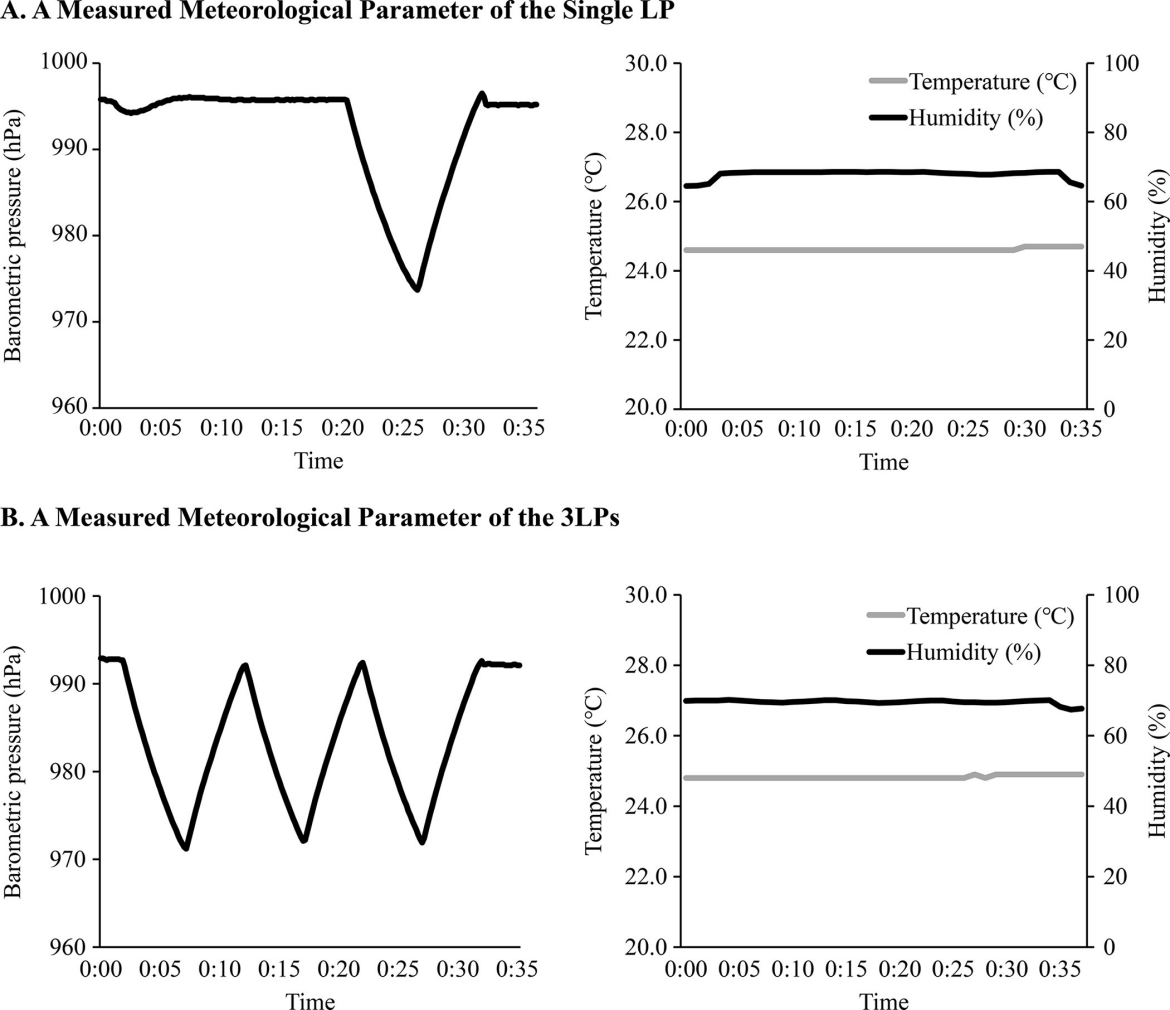
Meteorological parameters were measured in a climate chamber during the experiment. The lines show the values of barometric pressure, temperature, and humidity in the climate chamber measured in one of the behavioral experiments for the Single LP (A) and the 3LPs (B).

In this experiment, each of the 12 mice in both the single LP and 3LP groups was further subdivided into four groups of three mice each. Fig 4 shows samples of changes in the climate parameters during a single LP or 3LP. There were slight but not dramatic changes in temperature and humidity during the LP simulations. The climatic parameters of the experimental room at the beginning of each experiment are listed in Table 1. The behavioral tests for each protocol were conducted under similar climatic conditions.

**Table 1:** Climate parameters in each protocol for behavioral tests.

	Atmospheric Pressure (hPa)	Temperature (°C)	Humidity (%)
The Single LP	994.7±0.3	25.3±0.4	69.4±2.3
Control of the Single LP	994.1±0.4	24.6±0.1	66.4±2.8
The 3LPs	991.5±0.3	25.3±0.4	69.4±2.3
Control of the 3LPs	990.7±0.5	25.1±0.2	68.1±6.3

The values are presented as mean±standard deviation of the barometric pressure, temperature, and humidity in the climate chamber measured in the behavioral experiments.

**Fig 5:**
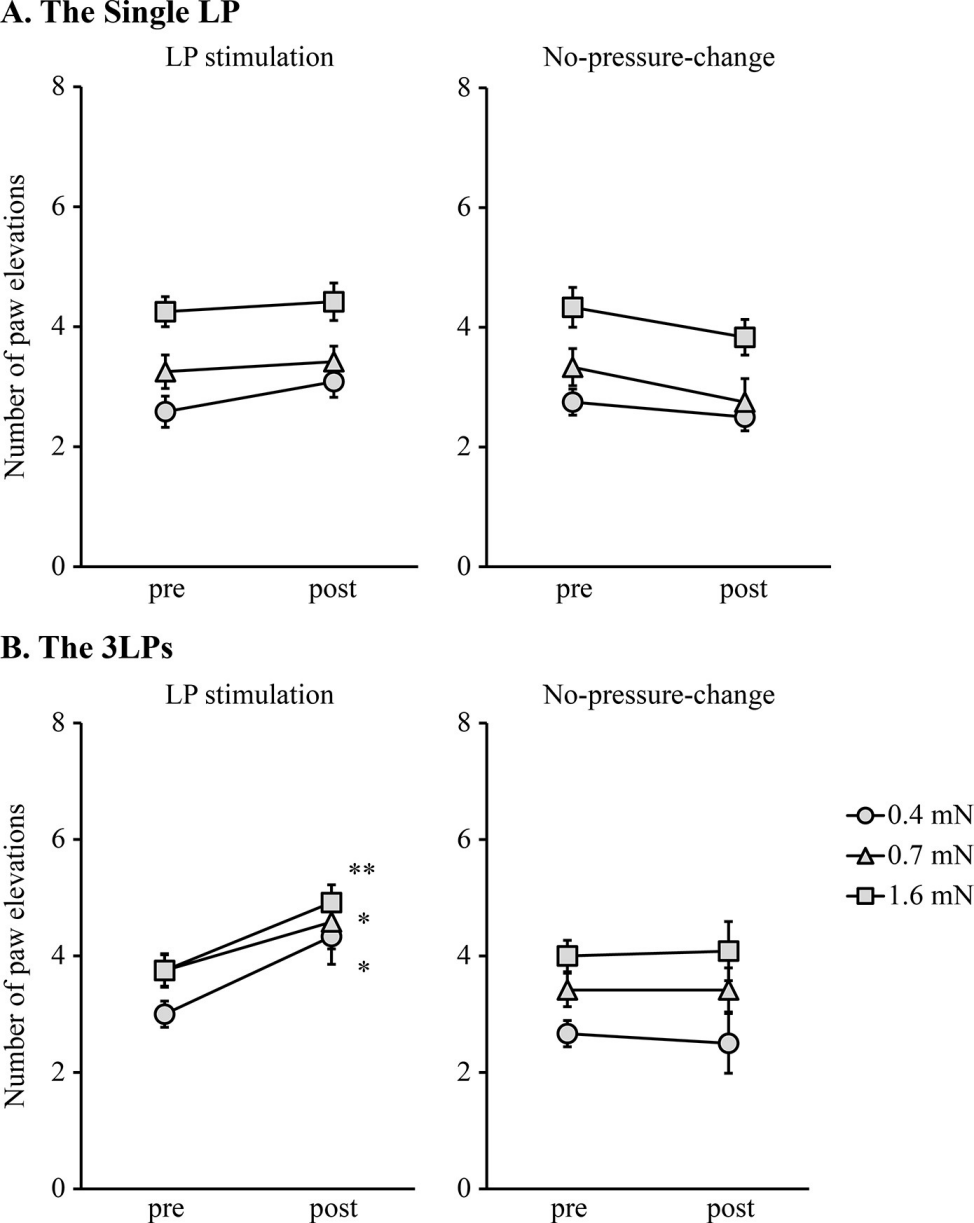
Effect of LP on mechanical hyperalgesia in CCI mice. The line plots show the number of paw elevations after mechanical stimulation by von Frey filaments in CCI mice exposed to a single LP (A), 3LPs (B), or their controls (n = 12 per group). “pre” indicates before exposure to the LP stimulation or no-pressure-change, and “post” indicates after exposure to the LP stimulation or no-pressure-change. *P < .05, ** *P* < .01 (Wilcoxon signed-rank sum test). Bar; SEM.

Fig 5 shows the number of hind paw elevations after mechanical stimulation, before and after LP exposure. Fig 5A shows the results of the Single LP and no-pressure-change protocols performed on the same mice on consecutive days. Regarding the number of hind paw elevations in any filament before and after LP stimulation, there were no significant differences in either the Single LP or the no-pressure-change protocol (Single LP, 0.4 mN, *P* = .193; 0.7 mN, *P* = .608; 1.60 mN, *P* = .627) (no-pressure-change protocol, 0.4 mN, *P* = .317; 0.7 mN, *P* = .083; 1.60 mN, *P* = .107).

Fig 5B shows the results of the 3LPs and no-pressure-change protocols performed on the same mice on consecutive days. In the 3LPs, the number of hind paw elevations after LP stimulation was significantly higher than that before LP stimulation in all filaments (0.4 mN, *P* = .007; 0.7 mN, *P* = .046; 1.60 mN, *P* = .039). However, there was no significant difference in the no-pressure-change protocol for any filaments (0.4 mN, *P* = .794; 0.7 mN, *P* = 1.000; 1.60 mN, *P* = .763).

The results for the 3LPs support previous findings that LP increases mechanical sensitivity in rat models with neuropathic pain. In contrast, no increase in mechanical hyperalgesia was observed after stimulation with a Single LP. The difference between the Single LP and 3LPs is the number of 20 hPa LP stimulations. Thus, these findings provide new evidence that the effects of LP stimulation on mechanical sensitivity can be accumulated and amplified according to the number of LP stimulations.

### Experiment 2. Effects of LP on plasma corticosterone levels in CCI mice

**Fig 6:**
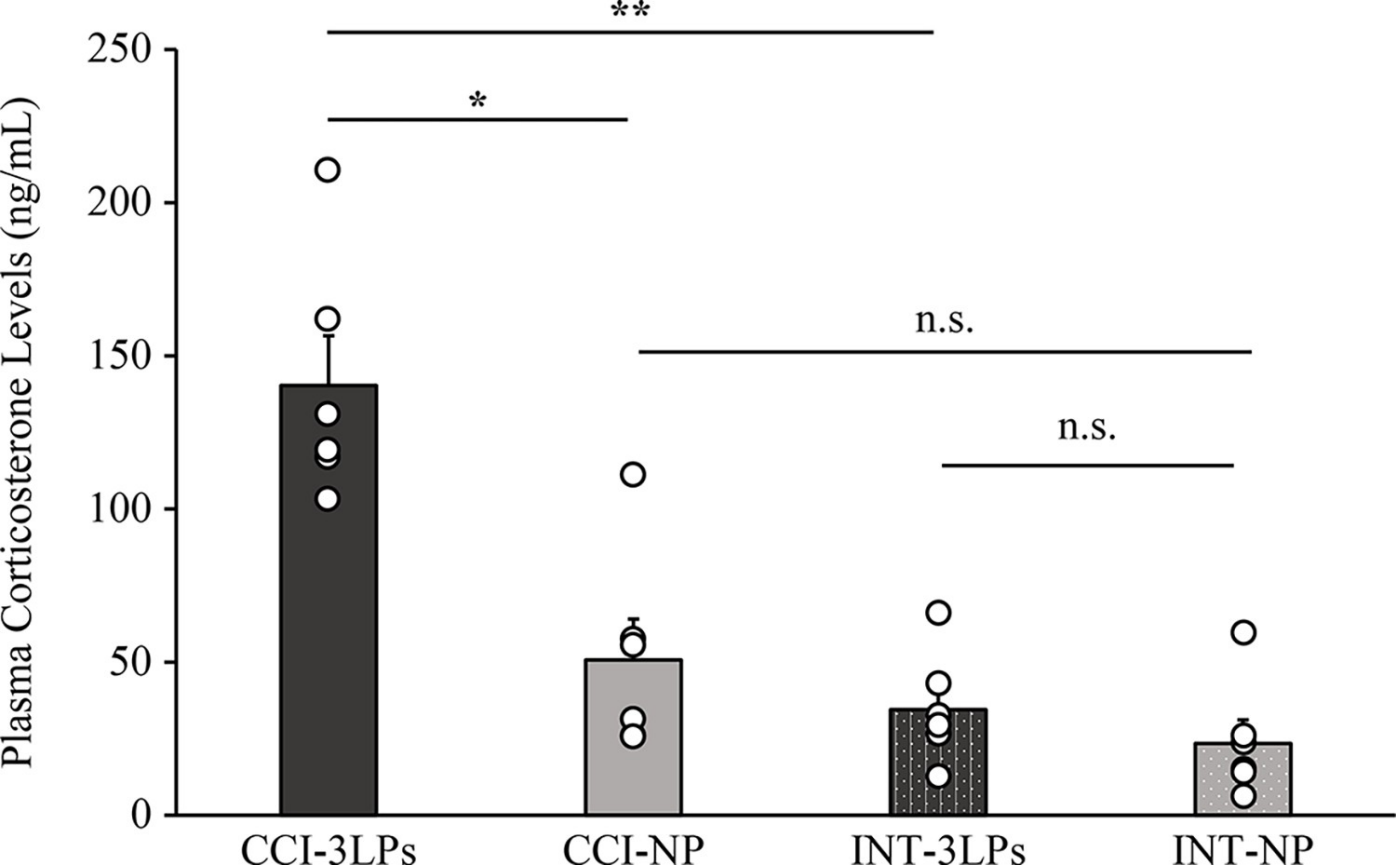
Effect of LP on plasma corticosterone levels in CCI and intact mice. Plasma corticosterone levels were measured by ELISA. The mean plasma corticosterone levels are shown in a bar graph with all values plotted. The graph on the left shows the results for CCI mice, and the graph on the right shows those for intact mice; n = 6 per group. *P < .05, ** *P* < .01 comparison of 3LPs and NP, and comparison of CCI mice and intact mice (Mann–Whitney U Test, Bonferroni-adjusted). n.s.; Not Significant. Bar; SEM.

Plasma corticosterone concentrations measured by ELISA, both with and without LP stimulation, are shown in Fig 6. A significant difference was observed among the groups (Kruskal–Wallis test: χ^2^ = 14.93, df = 3, p = .002). When comparing plasma corticosterone levels in both 3LPs and NP, the mean plasma corticosterone level in CCI-3LPs (140.3±16.3 ng/mL) was significantly higher than that in CCI-NP (50.72±13.4 ng/mL) (*P* = .017). However, in intact mice, there was no significant difference in the mean plasma corticosterone levels between INT-3LPs (34.5±7.4 ng/mL) and INT-NP (23.5±7.7 ng/mL) (*P* = .719). When comparing both intact and CCI mice, mean plasma corticosterone levels in CCI-3LPs were significantly higher than those in INT-3LPs (*P* = .008). In the no-pressure protocol, there was no significant difference in mean plasma corticosterone levels between intact and CCI mice (*P* = .260), although the levels in CCI-NP were slightly higher than those in INT-NP.

These results indicated that LP led to increased plasma corticosterone levels in CCI mice. In contrast, LP stimulation-induced corticosterone increases were not observed in intact mice. This likely reflects the fact that LP may be stressful only under chronic pain conditions in mice.

## Discussion

In the current study, increased sensitivity to mechanical stimulation in mice with neuropathic pain was observed with the three-time LP protocol but not with the single-LP protocol. This suggests that repetitive lowering of barometric pressure is an important meteorological factor that increases pain in mice. Moreover, the repeated lowering of barometric pressure led to elevated plasma corticosterone levels only in mice with neuropathic pain. Our data suggest that the barometric pressure effect on pain necessitates the presence of chronic pain, and that corticosterone may have an important relationship with LP-induced pain in mice.

The result was that only the 3LP protocol increased sensitivity to mechanical stimulation, indicating that the effect of a three-time LP on pain behavior lasts longer than that of a single LP. This finding implies that the effect of lowering barometric pressure on pain accumulates and lasts longer with repeated application. We have previously reported the rate and magnitude of hypotonic pressure changes in rats with neuropathic pain [15] but have not examined their cumulative effect. In human research, small, frequent changes in atmospheric pressure have been shown to affect cardiovascular diseases [25], migraines [1], and injuries [26], suggesting that frequent fluctuations in atmospheric pressure may be stressful for organisms. It is also reported that small pressure fluctuations of less than 1 hPa, which occur during the approach of typhoons, have been linked to weather-related pain in Japan [27]. However, due to the protocol used in our current experiment, which involves a far larger pressure change of 20 hPa repeated only three times, discussing the clinical significance of our results may be challenging. Therefore, our findings should be regarded as a preliminary indication of the potential impact of alterations in pressure frequency. To the best of our knowledge, this is the first animal report to demonstrate that repeated stimulation of LP can exacerbate neuropathic pain.

In the present study, we found no significant changes in sensitivity to mechanical stimulation in CCI mice before and after a single 20 hPa lowering in barometric pressure. However, previous reports have shown that lowering barometric pressure by 5 hPa or more aggravates sensitivity to mechanical stimulation in rats with neuropathic [13,15] or arthritic pain [14]. We believe that the results of the current study do not contradict those of the previous studies. First, earlier reports on behavioral tests for LP usually used rats, whereas this is the first report on mice, which may have caused some differences in the results. Additionally, the unique protocols for LP that differ from those reported previously [13–15] may be another reason for this discrepancy. In previous studies, pain behavior was tested while lowering the barometric pressure. However, in this study, the mechanical sensitivity was assessed after returning to barometric pressure at the beginning of each experiment. Given that the effect of LP on pain behavior is transient, it is possible that hypersensitivity subsided when barometric pressure returned. Additionally, we observed a slight, though not significant, enhancement in pain behavior after a single lowering of the barometric pressure. We observed an enhancement of hypersensitivity in the present study if measurements were taken during the lowering of the barometric pressure.

We selected these protocols in the present study for several reasons. The use of mice in this study made it technically difficult to measure pain behavior in the chamber during barometric pressure changes. Conversely, it has the advantage of blinding the examiner to the internal environment of the chamber. Another advantage is the ability to investigate the cumulative effect of LP and its impact on pain behavior. Therefore, our data strongly support previous findings that LP can worsen neuropathic pain [13].

Glucocorticoids are among the most common stress hormones. An increase in glucocorticoid levels is typically caused by the activation of the HPA axis as a stress response and is accompanied by an autonomic nervous system response. We previously reported that LP activates the autonomic nervous system in rats with neuropathic pain [17]. However, the relationship between reduced barometric pressure and changes in blood stress hormone levels remains unclear. Corticosterone is the primary glucocorticoid used in rodents. Plasma corticosterone levels in mice increase within minutes of stimulation and return to their original levels within three hours [22]. Thus, it may better demonstrate the effects of the accumulated impact of LP than the autonomic nervous system response, in which the response disappears quickly. The current study showed that the repeated lowering of barometric pressure enhances hyperalgesia in response to mechanical stimuli and leads to increased plasma corticosterone levels in mice with neuropathic pain. Our data revealed, for the first time, that LP directly or indirectly activates the HPA axis in mice with chronic neuropathic pain.

Although the pathway between LP and the increase in plasma corticosterone levels remains unclear, there are two possible explanations. First, the lowering of barometric pressure enhances spontaneous pain at the neuropathic site and elevates corticosterone levels. Second, lowering the barometric pressure itself is stressful for the organism. In the former explanation, local tissue changes at the pain site, such as dilation of blood vessels or activation of peripheral cells such as mast cells, could boost peripheral nerve activity. However, as discussed in a previous report [16], this mechanism is unlikely because the hind paw skin temperature did not change after exposure to lowered barometric pressure in rats.

However, the latter explanation is assumed to involve barometric pressure changes that stimulate the inner ear as a mechanical stimulus [20]. This change is then transmitted to a series of stress response systems, such as the limbic brain and HPA axis, leading to hypersensitivity to mechanical stimulation. This is based on a report that lowering barometric pressure induces vestibular neuron activation in the superior vestibular nucleus of mice [28]. Moreover, it has been reported that vestibular nuclei are morphologically connected to hypothalamic paraventricular neurons [29]. In this study, an increase in plasma corticosterone levels was observed only in CCI mice but not in intact mice. This finding suggests that LP serves as a stress stimulus only in mice with chronic neuropathic pain but not in intact mice. Additionally, in the no-pressure-change protocol, while there was no statistically significant difference, the plasma corticosterone levels in CCI mice were slightly higher than those in intact mice. This finding implies that the plasma corticosterone levels in CCI mice may have already been elevated due to chronic pain, even before exposure to LP stimulation. Chronic pain conditions can cause normally innocuous inputs to be perceived as aversion or even pain, including allodynia [30], photosensitivity [31], sound hypersensitivity [32], and olfactory hypersensitivity [33]. These changes are thought to reflect an adaptation to long-term pain conditions [34]. Acute stress leads to increased glucocorticoid secretion from the adrenal glands through a normal response of the HPA axis, resulting in systemic analgesia and anti-immune effects [35]. However, prolonged and frequent stress may contribute to a series of stress-related maladaptive states, recognized as allostatic loads involving variable systemic physiological and neurobiological dysfunctions [34]. The primary pathophysiology of the allostatic load is thought to involve excessive activation of the HPA axis [36] and abnormalities in brain networks [35,37], including morphological and functional plasticity changes in the hippocampus, amygdala, and prefrontal cortex [38–40]. Our study provides behavioral and endocrinological evidence that barometric pressure changes can stress the organism alongside other somatosensory inputs under chronic pain conditions. Furthermore, the observed increase in plasma corticosterone levels in the present study may indicate a series of maladaptive responses in the HPA axis associated with chronic pain.

According to the mechanisms described above, corticosterone may play an important role in the relationship between hyperalgesia, mechanical stimuli, and LP. Glucocorticoids have analgesic effects on acute stress, according to an earlier animal report [41]. Glucocorticoids are analgesic and anti-inflammatory agents used in the treatment of several inflammatory diseases. In contrast, glucocorticoids can exacerbate hypersensitivity to tactile stimulation by increasing glucocorticoid receptors in the spinal cord in rodents with neuropathic pain [42,43]. Based on the results of the current study, we could not determine whether corticosterone exacerbated pain or acted as an analgesic. No correlation was found between hypersensitivity to mechanical stimuli and plasma corticosterone levels in the present study (S1 Fig), suggesting that plasma corticosterone levels do not reflect the degree of hypersensitivity or pain in mice. Further research is required to determine the role of corticosterone in the relationship between barometric pressure and pain.

This study has several limitations. First, we did not investigate how changes in barometric pressure affected pain in terms of magnitude, rate, and frequency. The magnitude and rate of the LP were determined based on previous studies. Based on the performance of the machine used in this study, the maximum pressure change rate is 4 hPa/min. Therefore, we performed three 20 hPa pressure changes in five minutes, for a total of 30 minutes of LP exposure, and compared this to a single LP. Additional studies are required to clarify the magnitude, rate, and frequency of the pressure change parameters that affect pain sensitivity. As there are differences in somatosensory sensitivity between animal species, there may be differences between humans and rodents, potentially reducing the need for detailed rodent studies.

Second, we did not examine the duration of the post-CCI period required for the barometric pressure to affect pain. Although we tested three weeks after CCI, it is certainly possible that the influence of atmospheric pressure occurred in the early stages after CCI. However, based on our hypothesis, it may take a few weeks for these effects to manifest, as rodents typically require approximately three weeks for morphological and functional changes in the central nervous system and metabolic dysfunction, including alterations in the HPA axis due to chronic stress [39]. Given the likely minimal impact of atmospheric pressure on the body, any effect would be relatively small compared with the effects of surgery. This makes it challenging to detect the effects through behavioral tests during the acute postoperative period when the pain intensity is high.

Third, only female mice were used in this study. The results may be more stable in male mice because of the estrous cycle of female mice. Nevertheless, some studies reported that plasma or serum corticosterone concentrations in female mice did not differ regardless of the estrous cycle state [44,45]. Further research is required to confirm this hypothesis.

Fourth, we did not investigate how the plasma corticosterone level would be influenced by the Single LP protocol in the current study. Since an increase in mechanical sensitivity was not observed with Single LP, we did not include this protocol in the measurement of plasma corticosterone. As mentioned earlier, there may be no correlation between mechanical sensitivity and plasma corticosterone levels. Additionally, the aim of this study was not to determine the effect of plasma corticosterone levels on mechanical sensitivity. Further experiments could provide additional insights into the relationship between mechanical sensitivity and plasma corticosterone levels.

In conclusion, we showed that repeated lowering of barometric pressure increased sensitivity to mechanical stimulation and plasma corticosterone levels in mice with neuropathic pain. Our data provided strong evidence that barometric pressure is an independent environmental factor that affects pain. Furthermore, corticosterone may be a key factor in the mechanism by which LP aggravates pain. This study elucidated the detailed mechanism underlying the relationship between barometric pressure and pain.

## Supporting information

S1 Fig Correlation between corticosterone levels and mechanical hypersensitivity in CCI-3LPs. Each graph illustrates the Spearman correlation between the number of paw elevations (X) and plasma corticosterone levels (Y) for different thicknesses of von Frey filament. A positive correlation indicates that as the number of paw elevations (X) increases, plasma corticosterone levels (Y) also tend to increase. The Spearman correlation coefficients (ρ) and corresponding *P*-values are displayed on each graph.
(TIF)
