# Supplementary figures and images for: The effects of lowering barometric pressure on pain behavior and the stress hormone in mice with neuropathic pain
Source: PLoS One. 2025 Jan 17;20(1):e0317767. doi: 10.1371/journal.pone.0317767 (PMC11741632; doi:10.1371/journal.pone.0317767)

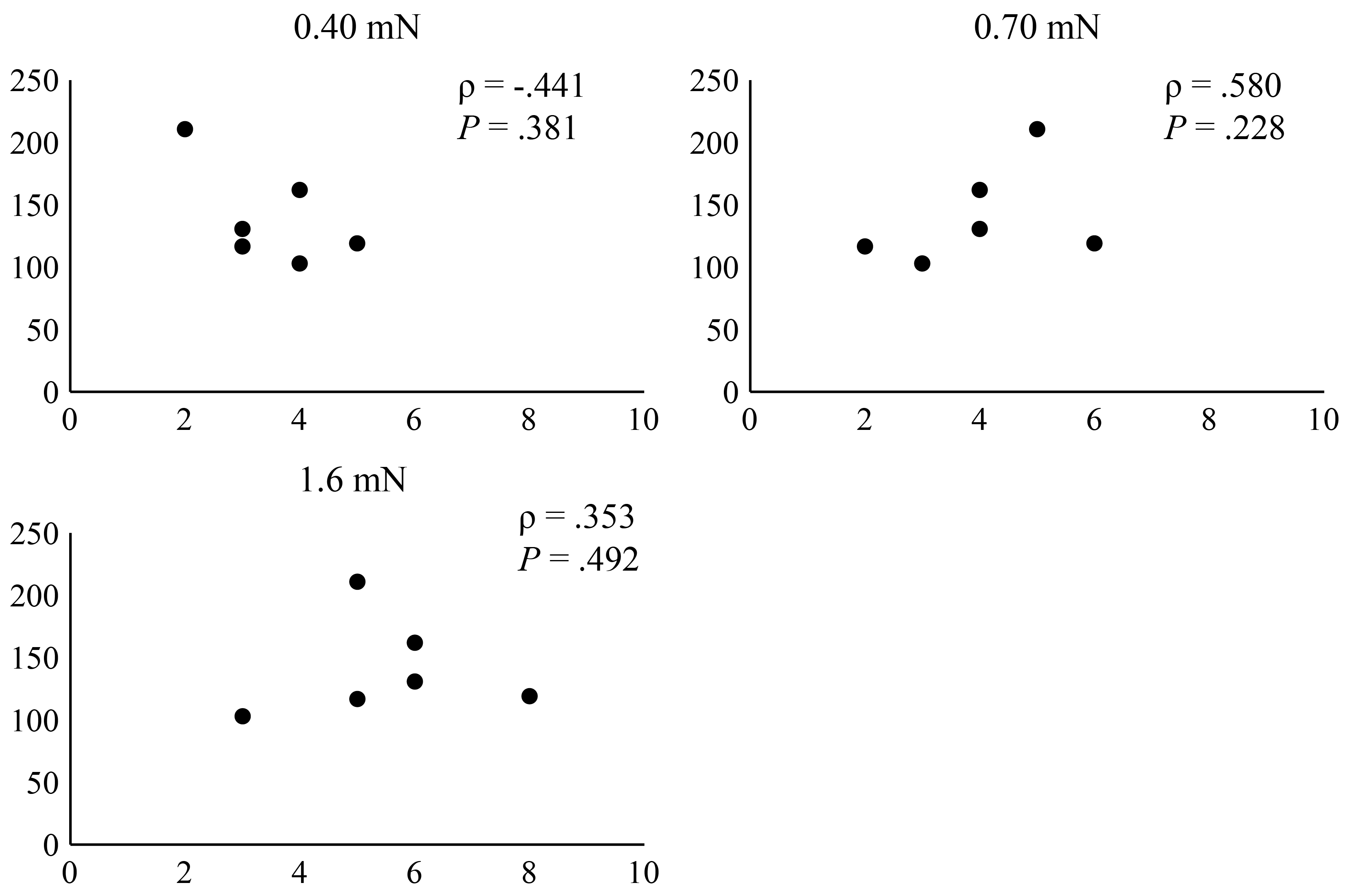

Supplement: S1 Fig — Each graph illustrates the Spearman correlation between the number of paw elevations (X) and plasma corticosterone levels (Y) for different thicknesses of von Frey filament. A positive correlation indicates that as the number of paw elevations (X) increases, plasma corticosterone levels (Y) also tend to increase. The Spearman correlation coefficients (ρ) and corresponding P-values are displayed on each graph. (TIF) [file pone.0317767.s001.tif]
